# Performance of water indices for large-scale water resources monitoring using Sentinel-2 data in Ethiopia

**DOI:** 10.1007/s10661-024-12630-1

**Published:** 2024-04-22

**Authors:** Mathias Tesfaye, Lutz Breuer

**Affiliations:** 1 Institute for Landscape Ecology and Resources Management (ILR), Research Centre for BioSystems, Land Use and Nutrition (iFZ), Justus Liebig University Giessen, Giessen, Germany; 2 Centre for International Development and Environmental Research (ZEU), Justus Liebig University Giessen, Giessen, Germany

**Keywords:** Copernicus data, Google Earth Engine, Remote sensing, SDG 6, Surface water

## Abstract

Evaluating the performance of water indices and water-related ecosystems is crucial for Ethiopia. This is due to limited information on the availability and distribution of water resources at the country scale, despite its critical role in sustainable water management, biodiversity conservation, and ecosystem resilience. The objective of this study is to evaluate the performance of seven water indices and select the best-performing indices for detecting surface water at country scale. Sentinel-2 data from December 1, 2021, to November 30, 2022, were used for the evaluation and processed using the Google Earth Engine. The indices were evaluated using qualitative visual inspection and quantitative accuracy indicators of overall accuracy, producer’s accuracy, and user’s accuracy. Results showed that the water index (WI) and automatic water extraction index with shadow (AWEIsh) were the most accurate ones to extract surface water. For the latter, WI and AWEIsh obtained an overall accuracy of 96% and 95%, respectively. Both indices had approximately the same spatial coverage of surface water with 82,650 km^2^ (WI) and 86,530 km^2^ (AWEIsh) for the whole of Ethiopia. The results provide a valuable insight into the extent of surface water bodies, which is essential for water resource planners and decision-makers. Such data can also play a role in monitoring the country’s reservoirs, which are important for the country’s energy and economic development. These results suggest that by applying the best-performing indices, better monitoring and management of water resources would be possible to achieve the Sustainable Development Goal 6 at the regional level.

## Introduction

Land surface water is one of the most decisive natural resources substantially changing in spatiotemporal terms, particularly because of land use and land cover dynamics as well as climate change. Land surface water encompasses streams, rivers, ponds, reservoirs, lakes, wetlands, and other inland water bodies (Pekel et al., 2016; Vandas, 2002). In spite of its limited extent, particularly in semi-arid and arid regions of the globe, land surface water is a resource for numerous human uses (e.g., drinking water supply, sanitation, and hygiene), ensures irrigation agriculture, or is used for hydropower production and industrial use. In addition, it maintains and supports biodiversity and provides essential and diverse ecosystem services (Brauman, 2015; Dudgeon et al., 2006; Zedler & Kercher, 2005). Surface water resources also play a vital role in the climate system and the hydrological cycle (Chahine, 1992; Tranvik et al., 2009). However, water-related ecosystems are fragile and vulnerable to anthropogenic impacts and climate change (Vörösmarty et al., 2000). The biodiversity of water-related ecosystems continues to deteriorate at an alarming rate (Collen et al., 2014). Associated with climate change are hydrological extremes such as flooding or droughts, and emerging water-related diseases, both leading to increasing losses of lives. Therefore, timely monitoring of regional-scale surface water resources is critical for policy and decision-making processes for its sustainable use and management (Giardino et al., 2010; Morss et al., 2005).

Global initiatives and policy frameworks like the SDGs and the Aichi Biodiversity Targets under the Convention on Biological Diversity (CBD) aim to ensure sustainable development of water resources, to reduce human impact, and prevent the loss of biodiversity (CBD, 2010; Griggs, 2013). Especially, the SDG 6 “Clean water and sanitation” and its target 6.6 “Protect and restore water-related ecosystems” emphasize the need to quantify its indicator 6.6.1 “Change in the extent of water-related ecosystems over time” (Dickens et al., 2017). Surface water monitoring is crucial for sustainable development, biodiversity conservation, ecosystem resilience, and people’s livelihoods. For this reason, improved monitoring of surface water resources on a large scale is needed, for example, by utilizing the latest advances of the Google Earth Engine with Sentinel-2 data for effective water management and decision-making processes. The implementation of water indices in this cloud computing system for improved water monitoring and management is in line with the broader goal of achieving SDG 6, thus contributing to both policy interventions and sustainable development efforts.

Thus, having the aforementioned potential conflicts in mind, this study contributed to quantifying the spatial distribution of the extent of water-related ecosystems, thereby ensuring its sustainable management in Ethiopia. Surface water plays a pivotal role, constituting more than 50% of the world’s water utilized for agricultural, domestic, and industrial purposes (OECD, 2015). This substantial reliance is largely attributed to lakes and reservoirs. Monitoring reservoirs is particularly important for providing early warning of drought, water, and food insecurity (Donchyts et al., 2022). Furthermore, effective reservoir monitoring serves as a deterrent to transboundary water conflicts and promotes international collaboration and also providing timely information by analyzing surface water dynamics as demonstrated for Turkey (Donchyts et al., 2022). The insights provided by surface water monitoring is needed for informed decision-making in environmental, agricultural, and urban water use, as for example shown for semi-arid Australia where it plays a crucial role in shaping water policy and management strategies (Tulbure et al., 2016). Overall, the integrated monitoring of surface water resources emerges as a support in addressing water challenges on a global scale, contributing not only to resource sustainability but also fostering international cooperation and informed governance.

**Fig. 1:**
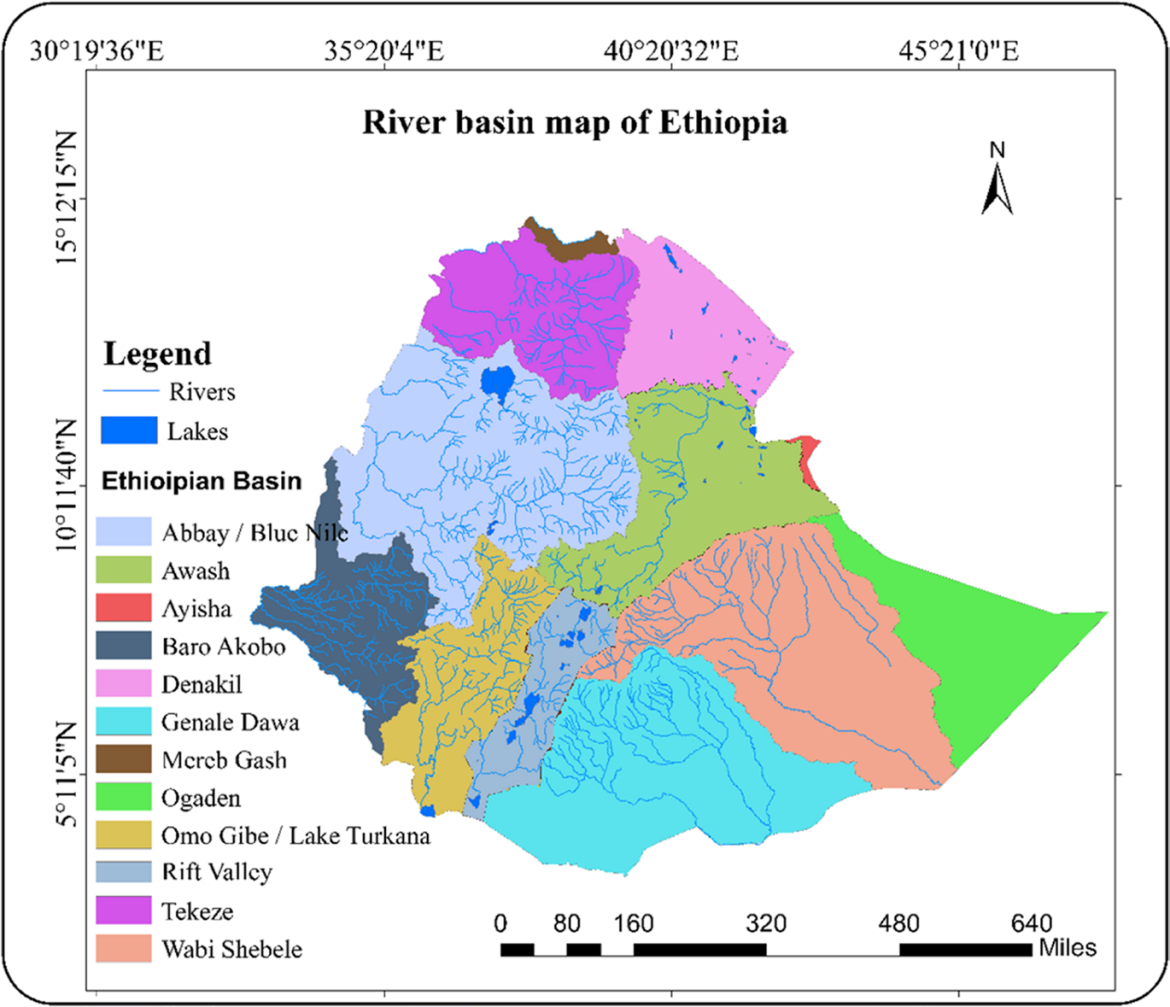
Main water bodies with basins of Ethiopia

Ethiopia has significant surface water resources with 12 major river basins, one lake basin and three dry basins (Fig. 1). A strong rainfall gradient separates the central and western highlands with abundant annual rainfall of up to 1200 mm from the arid southeast, east, and northeast, which receive 200 mm and below (Berhanu et al., 2014). Therefore, surface water resources are comparatively less available in the eastern part of Ethiopia (especially in the Awash basin) while the western part with the Abay (Blue Nile) river basin has large water resources. In contrast to the importance of water resources for human well-being and the country’s ecosystems, monitoring systems for the availability of surface water and hydrological flows are in poor condition and sometimes unreliable or malfunctioning (Dile et al., 2018). A country-wide continuous assessment of a water resource indicator as requested as part of SDG6 is not in place.

In contrast to in situ measurements, satellite remote sensing is an effective and efficient tool for monitoring and mapping surface water distributions. It allows covering a wide range of spatial and temporal scales because of its accessibility, repeatability, geospatial consistency, and global coverage (Fisher et al., 2016; Mueller et al., 2016). Satellite remote sensing plays a vital role in monitoring inland surface waters, particularly lakes and reservoirs. In the recent past, accurate surface water detection methods have been developed for a wide range of environmental conditions (Feyisa et al., 2014; Fisher et al., 2016), with particular benefit for methods and data fusion techniques using machine learning methods (Schmitt, 2020). Recent advances in remote sensing technology, characterized by high spatial, temporal, and spectral resolution, have contributed substantially to the monitoring of inland water bodies, with emphasis on their accurate detection (McCarthy et al., 2017), even though monitoring lakes and reservoirs is still a challenge for classification algorithms due to their characteristics. This challenge is for example shown for Lake Mead (USA), Nova Ponte reservoirs (Brazil), and Lake Williston (Canada) because of its temporal variability, various narrow ends, dynamic shape, and missing values by cloud or ice cover (Khandelwal et al., 2017). Therefore, this study tested surface water detection methods for the study area of Ethiopia using high spatial and temporal resolution in a cloud computing platform.

Google Earth Engine is such a cloud computing platform for global-scale analyses. It provides access to a range of satellite imagery, particularly Landsat and Sentinel-2 products and facilitates high-performance computing for social and environmental analysis, including water monitoring (Gorelick et al., 2017). The launch of the Sentinel program by the European Space Agency (ESA) as part of the Copernicus program was a breakthrough moment as it provided access to free, high-resolution images on a large scale for the first time. The band characteristics and spectral response methods of Sentinel-2 data are helpful to detect surface water bodies from the background (Jiang et al., 2021). Thus, in this study, Google Earth Engine was employed to process the potential of water index methods and selected the best-performing indices for surface water detection using multi-spectral satellite imageries of Sentinel-2 data.

Surface water can be detected using multi-spectral satellite imageries on the base of the significantly lower infrared reflectance of water compared to other land cover types. Hence, based on the peculiarities of the Near-Infrared/Short-Wave Infrared (NIR/SWIR) domain, numerous approaches have been developed for extracting surface water from remote sensing imageries.

The water indices method is a common classification method using multi-bands (Fisher et al., 2016). It is easy to use and quick to calculate (Ryu et al., 2002). Further indices include the normalized difference water index (NDWI) (McFeeters, 1996), the modified normalized difference water index (MNDWI) (Xu, 2006), and the land surface water index (LSWI) (Xiao et al., 2002). All these indices have been widely used in Landsat 5 Thematic Mapper (TM) and Landsat 7 Enhanced Thematic Mapper (ETM+) imagery analyses. They are easy to compute and rely only on two input bands. Feyisa et al. (2014) developed the automatic water extraction with no shadow (AWEInsh) using four bands (green, NIR, SWIR1, and SWIR2) (Feyisa et al., 2014). The AWEIsh additionally utilizes the blue band. AWEIsh is designed for shadows from mountains, buildings, and clouds. The sentinel water index (SWI) is computed using red-edge1 and SWIR1 bands of Sentinel-2 (Jiang et al., 2021). The water indices methods have the benefits of robustness, quick detection, and high accuracy in large-scale surface water detection (Li et al., 2013; Wang et al., 2021; Feyisa et al., 2014). Each water index was designed and previously tested using Landsat imagery, except for the SWI. Therefore, this study evaluated the performance of each water index method using Sentinel-2 data in Ethiopia.

A few studies have been carried out to monitor surface water resources in Ethiopia using water indices with Landsat imagery. For example, NDWI was used for surface water extraction and change detection in the Central Rift Valley region of Ethiopia, specifically Abjata, Shala, and Langano Lakes (Sisay, 2017). Another study demonstrated the use of NDWI to monitor surface water in Chelekleka, Crafty, Coke, and other lakes (Sathianarayanan, 2018). An evaluation of the performance of three water indices was carried out at Lake Zeway, which confirmed that the AWEI performed better than the MNDWI and NDWI in detecting surface water bodies (Asfaw et al., 2020). However, Ethiopia faces challenges related to limited spatio-temporal information on water resources at the national level. This lack of information is particularly problematic given its importance for effective water management and decision-making. It is also a key challenge for achieving the SDG 6 indicators. Ethiopia’s diverse landscapes require the selection of appropriate water detection and monitoring techniques tailored to local conditions. Such an analysis would also increase the knowledge of remote sensing applications for water resources in different geographical settings. The performance of a wide range of water index methods has not been tested on the country level of Ethiopia, and a comparative performance analysis for the more recent Sentinel-2 data is missing.

Choosing the best index for large-scale surface water detection is difficult due to inconsistent results obtained from various indices and unstable threshold values to differentiate water from non-water, which is changing with location and scene (Ji et al., 2009). Therefore, this study sets out to identify optimal thresholds for large-scale assessment. In Ethiopia, surface water detection is challenging at a large scale due to the labeled water and non-water feature datasets being limited. Hence, the objective of this work was to demonstrate the potential of water index methods and to select the best-performing indices for detecting surface water using high-resolution and multi-temporal Sentinel-2 data at the country scale. The indices were calculated using the Google Earth Engine. The spatial distribution of water resources in Ethiopia and monitor surface water resources in relation to the fulfillment of SDG 6 are investigated.

## Materials and methods

### Description of the study area

Ethiopia’s diverse topography ranges from the depths of the northeastern Afar Depression at 116 m below sea level to the towering heights of the Ras Dashin Mountains at about 4600 m above sea level. This has created a diverse of agro-ecological zones, climates, and soil compositions. The agro-ecological zones are categorized into six major groups (MoA, 2000), which include arid, semi-arid, sub-moist, moist, sub-humid, and humid zones. Ethiopia’s climate is traditionally divided into five distinct zones, based on altitude and temperature: “Wurich” (cold to moist), “Dega” (cool to humid), “Weynadega” (cool sub-humid), “Kola” (warm semi-arid), and “Berha” (hot arid). Average temperature variations are significant, ranging from 5 °C in the highlands to about 40 °C in the lowlands (Gebrechorkos et al., 2023). Rainfall patterns show considerable spatial and temporal variation. It ranges from 100 mm/yr in the arid northeastern lowlands to 2500 mm/year in the abundant southwestern highlands. The country experiences two main rainy seasons: the “Belg” from March to May, which is characterized by light rainfall, and the “Kiremt” from June to September, which is the main rainy season.

Geologically, Ethiopia’s landscape is shaped by three main structural units, i.e., Precambrian basement, Paleozoic and Mesozoic sediments, and Cenozoic volcanites and sediments (Hurni et al., 2007). These geological formations contribute to the diverse topography and soil characteristics that are observed throughout the country. Soil composition adds another layer of diversity to Ethiopia’s agricultural landscape. More than half of the country’s arable land is covered by nitosols (23%), cambisols (19%), and vertisols (18%) (Dubale, 2001). Nitosols and cambisols dominate the highlands west of the Rift Valley and the Afar region, whereas xerosols and yermosols dominate the northern and southern regions, respectively. Agriculture is the backbone of the Ethiopian economy, with crop production being the main economic activity. It contributes about 40% of the country’s gross domestic product (Jimma et al., 2024).

Ethiopia is endowed with substantial surface water resources and 12 major river basins. (Fig. 1). A majority of Ethiopia’s rivers experience seasonal variation and approximately 70% of the total runoff occurs between June and September (FAO, 2016). Four of Ethiopia’s river basins, namely Abbay or Blue Nile, Baro-Akobo, Tekeze, and Mereb which are parts of Nile basin, cover 33% of the country and drain the northern, central, and western parts (FAO, 2016). In the eastern part of Ethiopia, surface water resources are limited since almost no perennial rivers are found below 1500 m a.s.l. Three of the main basins (Aysha, Dinakle, and Ogaden) are mainly dry with no permanent discharge (Berhanu et al., 2014). Ethiopia’s 12 major lakes cover around 7300 km^2^. Lake Tana is the largest lake in the Abay Basin. It is the main water source of the Abay River. Most other lakes are saline and located in the Rift Valley.

### Sentinel-2 data and pre-processing

Sentinel-2 data from December 1, 2021, to November 30, 2022, were used. Images were acquired by the Copernicus program in the Earth Observation program of the European Union, which provides multi-spectral data in the visible, near-infrared, and shortwave infrared parts of the spectrum, a total of 13 bands. The tiles cover 100 by 100 km^2^ with a spatial resolution of 10 m (bands blue (B2), green (B3), Red (B4), and NIR (B8), 20 m (red edge 1 (B5), red edge 2 (B6), red edge 3 (B7), red edge 4 (B8a), SWIR 1 (B11), and SWIR 2 (B12) and 60 m (aerosols (B1), water vapor (B9), and cirrus (B10)) for one of the most widely available Level-1C standard product. The temporal resolution or the revisit frequency of each individual Sentinel-2 satellite is 10 days and the combined constellation revisit is 5 days.

The Sentinel-2 imagery was collected using the Sentinel-2 Surface Reflectance product *COPERNICUS/S2_SR*. However, Sentinel-2 imagery covers all of Ethiopia only in December, March, and January among all months. The images were then filtered based on the specified time range using the *filterDate* function. The dataset was further refined spatially by filtering based on a designated region of interest using *filterBounds(roi)*. Subsequently, images with a cloudy pixel percentage exceeding 20% are excluded using *ee.Filter.lt(‘CLOUDY_PIXEL_PERCENTAGE’, 20)*. Clouds were then masked out using a custom cloud-masking function through the cloud/shadow mask function in Google Earth Engine. Processing was performed in all spectral bands and the cloud mask detected cloud-free and cloudy pixels, including both dense clouds and cirrus clouds. Thus, the processing steps combining cloud and cirrus masking and atmospheric correction function in the Google Earth Engine contributed to cloud-free pixels and atmospheric correction of Sentinel-2 imagery. Lastly, the median value for each pixel across all filtered images was calculated using the *median()* function, resulting in a single composite image that effectively aggregates Sentinel-2 observations over the specified temporal and spatial extent.

### Surface water detection

Seven water indices (Table 1) were calculated in the Google Earth Engine computing system. All indices have originally been developed using Landsat images except for the SWI. Various mechanisms have been used to obtain optimal thresholds for distinguishing water from land. We applied the optimum threshold value for the automated water extraction index by considering the error of commission and omission. The threshold ranges between − 1 and + 1, and a default threshold of zero does not represent the highest water detection accuracy (Feyisa et al., 2014). The selection of the optimum threshold involves an iterative trial and error process (Acharya et al., 2018). At the beginning, an initial threshold of default value zero for the water index was set and applied to the dataset. The classification accuracy was then assessed by incrementally adjusting the threshold until the highest overall accuracy values were achieved. This approach ensured optimal differentiation between water and land.

**Table 1:** Water index methods

Index	Index name	Source	Equation
NDWI	Normalized Difference Water Index	McFeeters (1996)	$\frac{\left(Green-NIR\right)}{\left(Green+NIR\right)}$
MNDWI	Modified Normalized Difference Water Index	Xu (2006)	$\frac{\left(Green-SWIR1\right)}{\left(Green+SWIR1\right)}$
AWEInsh	Automated Water Extraction Index non-shadow	Feyisa et al. (2014)	(4*(Green-SWIR1))-(0.25*NIR+(2.75*SWIR2))
AWEIsh	Automated Water Extraction Index shadow	Feyisa et al. (2014)	(Blue+2.5*Green-1.5*(NIR+SWIR1)-0.25*SWIR2)
WI	Water Index	Fischer et al. (2016)	(1.7204+171*Green+3*Red-70*NIR-45*SWIR1-71*SWIR2)
SWI	Sentinel Water Index	Jiang et al. (2021)	$\frac{\left(RedEdge1-SWIR1\right)}{\left(RedEdge1+SWIR1\right)}$
LSWI	Land Surface Water Index	Xiao et al. (2002)	$\frac{\left(NIR-SWIR1\right)}{\left(NIR+SWIR1\right)}$

### Evaluation of water indices

Accuracy assessments were carried out to determine whether or not the results of surface water extraction are acceptable. In this study, qualitative (visual) and quantitative assessments were undertaken. In the visual assessment, the magnitude of continuousness and the smoothness of the boundary of water bodies were assessed and cross-checked with reference data. During classification, 80% of the samples were used for calibration and training, while the remaining 20% were kept for validation (Elith et al., 2011). For validation, sample points were collected from different locations and considered seasons from December 1, 2021, to November 30, 2022, using georeferenced samples from Sentinel-2 and coincident high-resolution imagery and OpenStreetMap data in Google Earth Engine.

For a coverage of < 4000 km^2^ per land cover and less than 12 classes of land cover, Lillesand et al. (2008) recommend at least 50 samples for each map class. However, sample point sizes vary in the literature. For example, to assess accuracy by visual interpretation using Sentinel-2 data, Xia et al. (2019) selected 1000 sample points for water and non-water in the Huai River Basin and Wang et al. (2020) collected 1500 points each in the Hetao Plain. For surface water extent estimation in France using 2 years of Sentinel-2 data, a total of 4800 sample points were evaluated by visual interpretation in the Google Earth Engine platform (Yang et al., 2020). Hence, in this study, we collected a total of 4680 sample points for the validation as a whole. Specifically, 2340 samples were taken from water bodies. The remaining 2340 samples included non-water bodies from a wide range of land cover types such as agricultural land, forest, grassland, built-up areas, and bare land. These sample points were collected throughout the entire study area using a stratified random sampling approach. Water bodies were additionally stratified by size and type to ensure that the assessment was representative of the diversity of water bodies present in the study area. Spatial stratification also improves the assessment of the classification accuracy of remote sensing data (Dong et al., 2022). Spatial autocorrelation examines how pixels that are close together are more similar than those that are far apart (Karasiak et al., 2022), resulting in falsely high precision metrics (Roberts et al. 2017; Meyer et al. 2019). Thus, features were separated by a minimum distance of 500 m to reduce spatial autocorrelation (Cabra-Rivas et al., 2016). This minimum distance for separating features ensures that they are spatially independent, reducing the risk of biased accuracy results and providing a more objective assessment of algorithm’s performance. The quantitative assessment was carried out using the 4680 feature sample points. Indicators of evaluation include producer accuracy, user accuracy, and overall accuracy.

Confusion matrices, the common method of describing the accuracy of the classification (Lillesand et al., 2008), were used to compare the reference data and the corresponding classification outputs on a category-by-category basis. User accuracy is computed by dividing the number of correctly classified pixels in each category by the total number of pixels that are classified in that category (the row total), which is known as the specificity or true negative rate, and the complement of the commission error (Lillesand et al., 2008). Whereas the producer accuracy is obtained by dividing the number of correctly classified pixels in each category (on the major diagonal) by the number of training set pixels used for that category (the column total), which is known as the sensitivity or true positive rate, and the complement of the omission error. The user’s accuracy, producer’s accuracy, overall accuracy, error of omission and error of commission were computed including the confusion matrix for the classification in Google Earth Engine computing. Finally, based on the aforementioned indicators of accuracy, the better-performing indices were selected and then used to quantify the spatial distribution of surface water resources in Ethiopia.

## Results

### Surface water detection

**Fig. 2:**
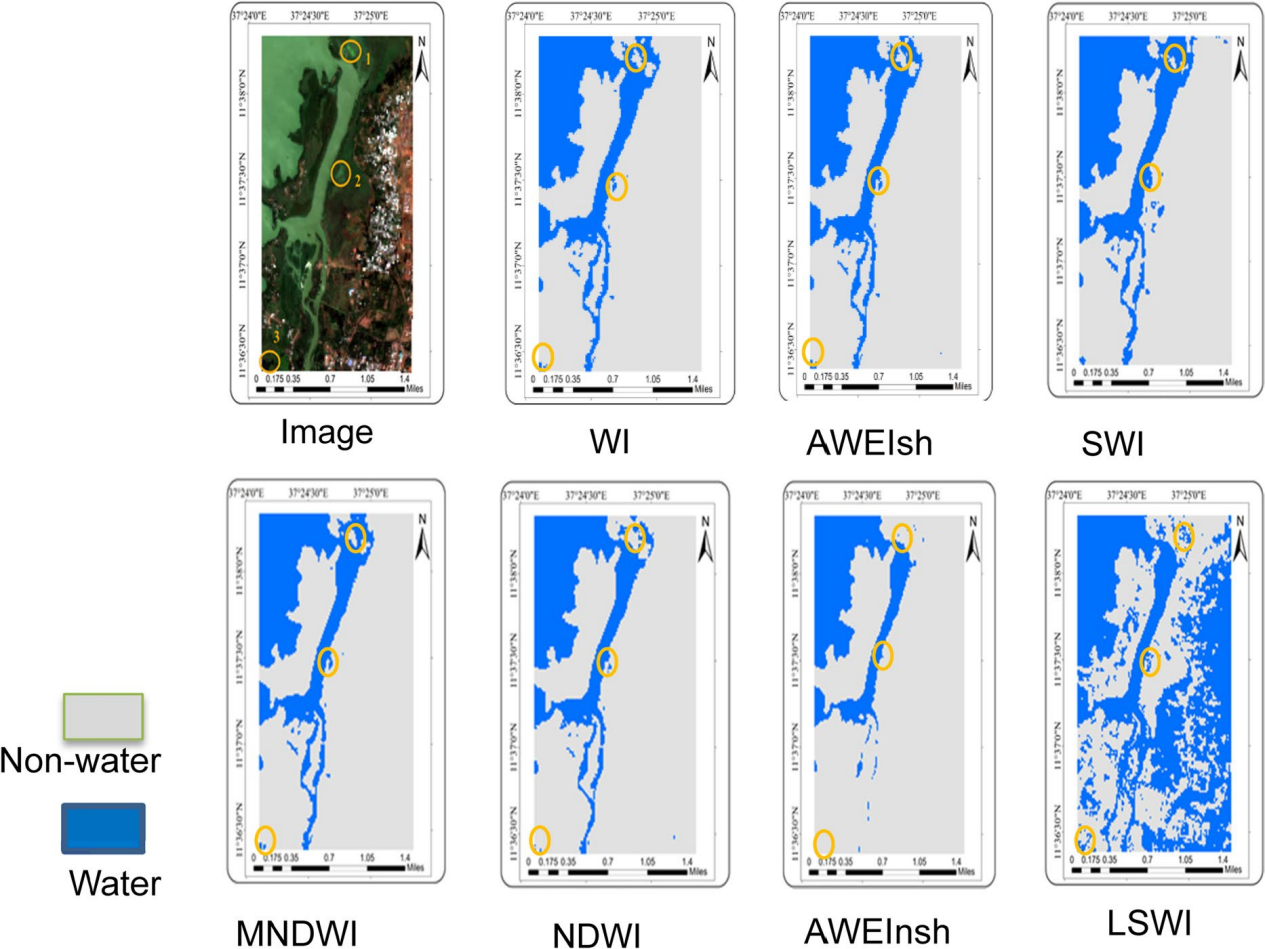
Specific map section for visual assessment and comparison of water indices outputs for part of the lake Tana area in Bahir Dar

**Fig. 3:**
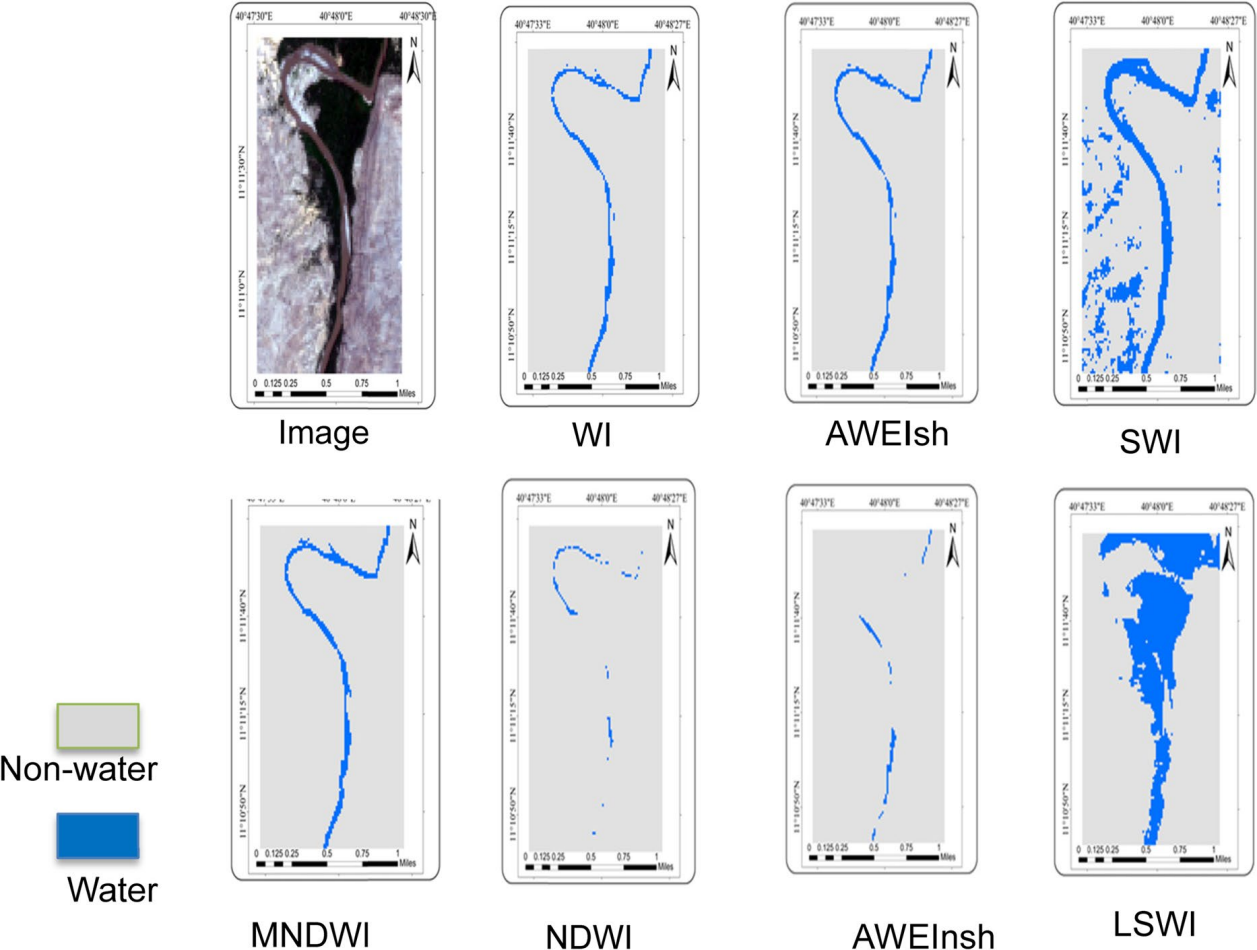
Specific map section for visual assessment and comparison of water indices outputs for the Awash river reach in Adaytu, Afar

**Fig. 4:**
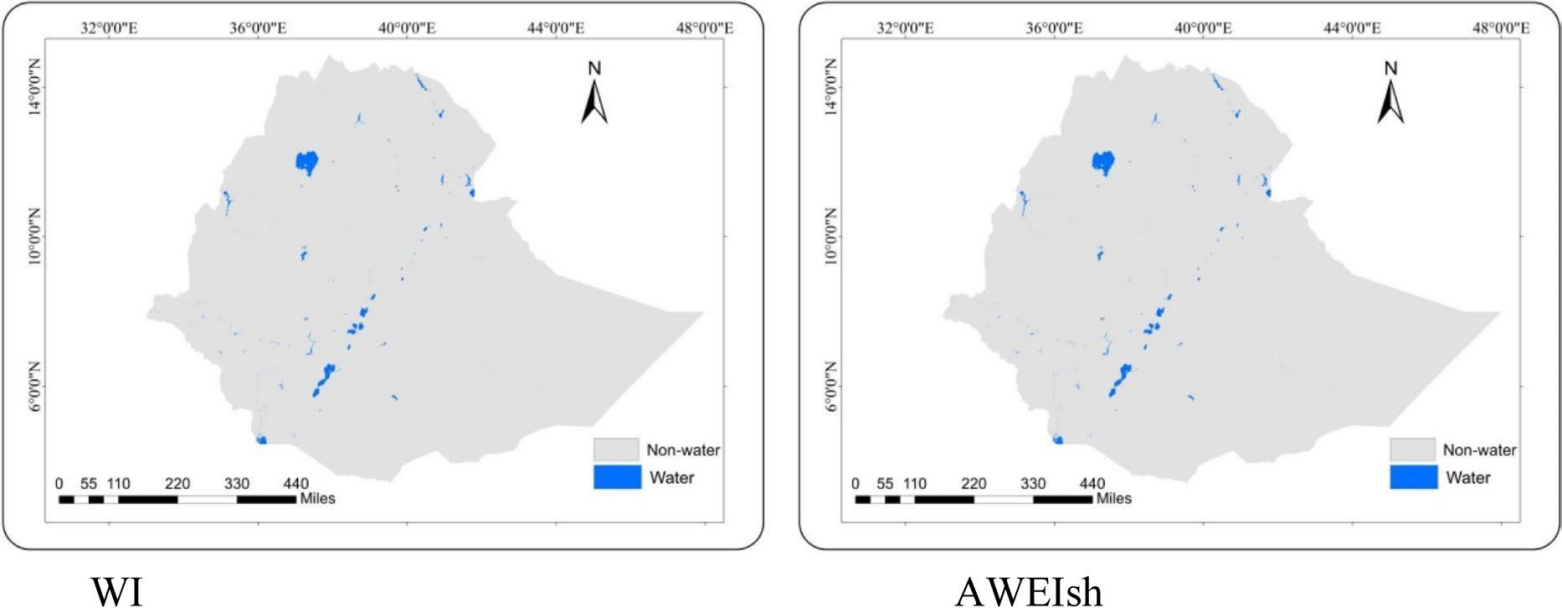
Ethiopia surface water coverage map using best-performing water indices

The results of water detection using seven indices for specific locations and the whole of Ethiopia were analyzed by visual inspection (Figs. 2, 3, and 4). The optimum threshold values that separate water from non-water pixels were selected for NDWI (0.06), MNDWI (0.14), AWEIsh (0), AWEInsh (0.04), WI (0.02), SWI (0.03) & LSWI (0.12). WI and AWEIsh were relatively better at detecting surface water where the surrounding areas are vegetated areas and urban areas (Fig. 2, #1 and #3). SWI was less good as there was some misclassification as water bodies (Fig. 2, #1 and #2). NDWI was relatively less effective than MNDWI because of some unclassified water pixels (Fig. 2, #1 and #3). In this case, AWEInsh only detects large water bodies, and LSWI was also unable to detect water bodies correctly. In flat areas, WI and AWEIsh were more effective to detect rivers compared to other water indices (Fig. 3). MNDWI was also superior in these flat and less vegetated areas, while NDWI was less able to map the rivers. SWI detected the river but misclassified non-water pixels as water pixels (Fig. 4). AWEInsh and NDWI were unable to identify rivers and small water bodies. Therefore, in this large-scale water detection experiment using Sentinel-2 data in Google Earth Engine, the WI and AWEIsh were most effective for surface water detection from a visual inspection point of view.

### Performance evaluation of water indices

The water producer’s accuracy ranged from 32 to 96%, whereas non-water producer’s accuracy ranged from 84 to 97%. All indices, except LSWI, had a satisfying classification accuracy > 85% with regard to the overall accuracy, producer’s accuracy, and user’s accuracy (Table 2). The WI achieved the highest accuracy with an overall accuracy of 96%. All other indicators, except for the LSWI, had only slightly worse performance criteria in terms of user's and producer’s accuracy for water, as well as overall accuracy. The difference in accuracy between the LSWI and the other indices was striking. This index was the least-performing one with an overall accuracy of 78%. Overall, the AWEIsh and particularly WI outperform the other indices regarding accuracy indicator performances depicted by the heat map (Table 2), whereby it must be said that the differences between six of the seven indices were in part only marginal. Together with the information from the visual assessment, we concluded that in this study, the WI and AWEIsh were the most accurate water indices using the high and multi-temporal resolution of Sentinel-2 data for surface water detection in Ethiopia.

**Table 2:** Heat map of classification accuracies of water indices

	Producer accuracy	Error of omission	Producer accuracy	Error of omission	User accuracy	Error of commission	User accuracy	Error of commission	Overall accuracy
	Water (%)		Non-water (%)		Water (%)		Non-water (%)		(%)
NDWI	85	15	92	8	90	10	92	8	90
MNDWI	92	8	93	7	93	7	93	7	92
AWEInsh	94	6	95	5	93	7	95	5	94
AWEIsh	95	5	96	4	94	6	97	3	95
WI	96	4	97	3	96	4	96	4	96
SWI	91	9	94	6	93	7	94	6	93
LSWI	32	68	84	16	36	66	81	19	78

Finally, the surface water coverage was calculated using the seven indices across Ethiopia. WI, AWEIsh, and MNDWI indices extracted more or less similar surface water coverages of 82,650, 86,530, and 88,160 km^2^, respectively. Slightly deviating was the coverage estimated by NDWI (79,650 km^2^). Completely different were the remaining coverages derived by the AWEInsh (51,800 km^2^), SWI (111,500 km^2^), and LSWI (207,520 km^2^).

## Discussion

The special spectral properties of water are utilized when detecting surface water and non-water surfaces. Water absorbs light above 700 nm, particularly in the NIR and MIR bands. Thus, water bodies can be identified based on their unique absorption characteristics by analyzing the reflectance spectrum of different surfaces. This analysis goes beyond color information and is based on how water behaves in the electromagnetic spectrum. In the visual assessment, the results showed that the WI and AWEIsh indices had a superior accuracy in surface water detection. They were more effective to detect surface water in urban and vegetated areas, despite that the detection in such areas is commonly difficult due to shadows (Feyisa et al., 2014). Shadows in urban areas are often misclassified as water bodies, as they have similar low reflectivity characteristics to water bodies (Liu et al., 2022). Fischer et al. (2016) stated that WI and AWEIsh performed best, whereas the MNDW and AWEInsh performed less accurately, and NDWI the least. In this study, MNDWI was more effective than NDWI, AWEInsh and SWI in detecting rivers and small water bodies. The MNDWI is an improved version of the NDWI in that it uses the SWIR band instead of the NIR band that is used in the NDWI to normalize the water and vegetation indices (Xu, 2006). NDWI was less sensitive to small water bodies and rivers than MNDWI and was also affected by noise from urban and vegetated areas. This is due to the spectral signature response of water bodies, which is less sensitive in the NIR band of the NDWI than in the MIR band of the MNDWI (Xu, 2006). In contrast, the MIR band in MNDWI can enhance the contrast between water and surrounding land, making it more effective at detecting smaller water features that may be missed by NDWI. In addition, the MIR band used in MNDWI is less sensitive to vegetation and less affected by noise from urban areas than the NIR band used in NDWI. As a result, the NDWI was unable to detect part of the Awash River and small water bodies in the Lake Tana area (Figs. 2 and 3). The contrast between water and land is acceptable for MNDWI, although it is less accurate than the WI and AWEI in detecting small water bodies or small streams (Liu et al., 2022). SWI was better than NDWI, AWEInsh, and LSWI for the detection of the same features. However, it was challenged by the elimination of shadow noise from surrounding non-water features. In general, SWI is better than NDWI at detecting wide river channels (Jiang et al., 2021).

The results showed that the WI and AWEIsh were the most accurate water indices. A similar finding was observed in four case studies in Switzerland, Ethiopia, South Africa, and New Zealand, where AWEIsh achieved water user’s and producer’s accuracies of 96–99% and 91–99%, respectively (Feyisa et al., 2014). Similarly, AWEInsh was also detected in a shadow-free image from Denmark with water user’s and producer’s accuracy of 98% and 92%, respectively (Feyisa et al., 2014). AWEI with Google Earth Engine is a quick and robust method for surface water monitoring (Nguyen et al., 2019). Overall accuracies of WI (98%) were also outperforming other indices (MNDWI 97%, NDWI 95%) in image analyses from eastern Australia (Fisher et al., 2016). WI achieved an overall accuracy of 96% and AWEIsh also performed well with an overall accuracy of 96% (Liu et al., 2022). In this study, LSWI performed worse due to the limitation of the index in interpreting Sentinel-2 data in this study area. It was also heavily influenced by the background noise of non-water features in the study area.

Overall, the performance evaluation criteria in this study are common and acceptable for evaluating the results of surface water detection using water indices (Feyisa et al., 2014; Fisher et al., 2016). The results present were even slightly higher than those of previous works, which range from 90 to 96% in overall accuracy, except for LSWI. This is perhaps due to the use of relatively higher spatial and spectral resolution of the Sentinel-2 data with Google Earth Engine processing. In addition, Sentinel-2’s band characteristics and spectral response methods are likely more effective than Landsat imagery at detecting water bodies from the background (Jiang et al., 2021).

**Fig. 5:**
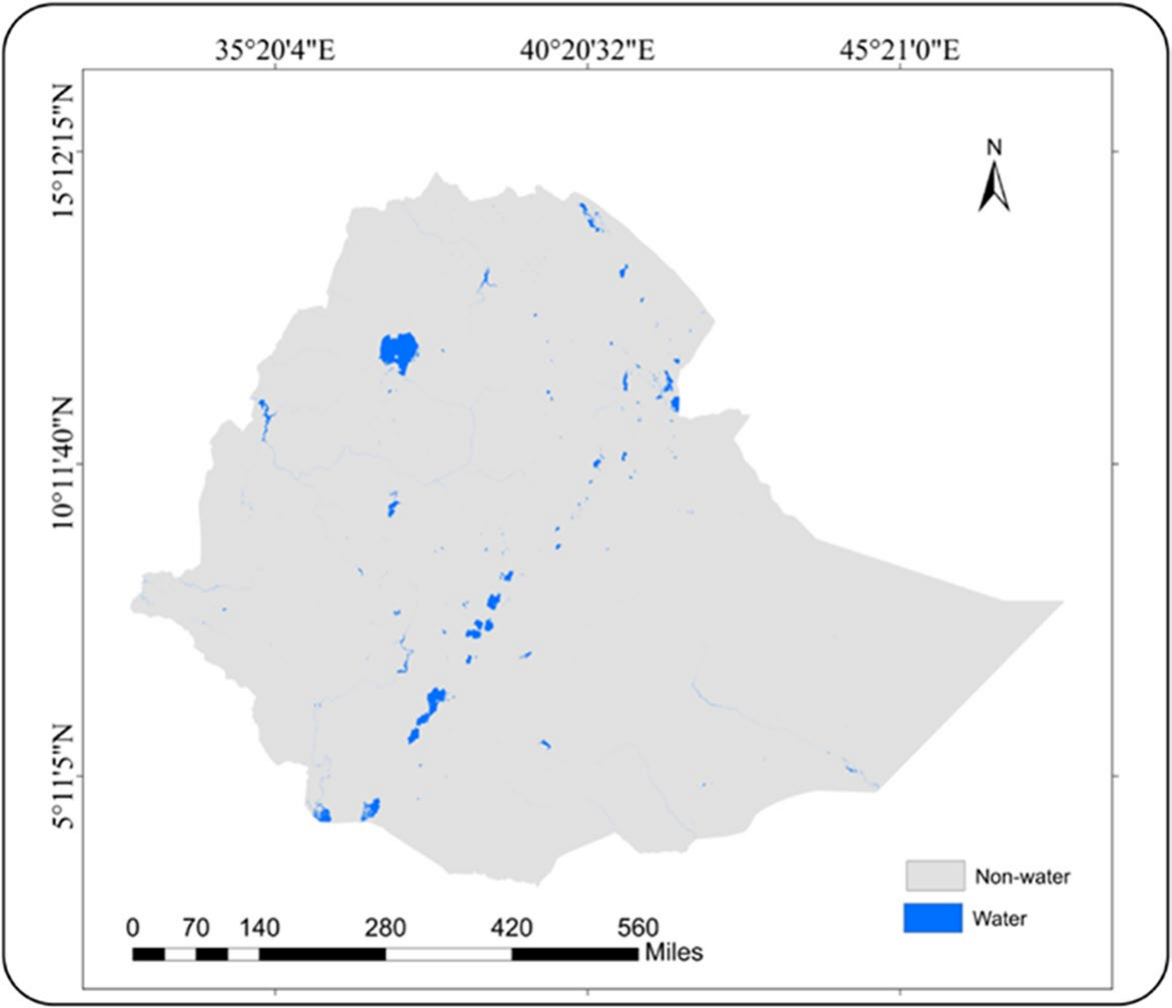
Extracted map of surface water from GSW dataset

In this study, the better-performing WI and AWEIsh predicted 82,650 and 86,530 km^2^ of surface waters in Ethiopia, respectively. A slightly higher coverage of 91,056 km^2^ was obtained by extracting the Global Surface Water dataset which was developed by the European Commission’s Joint Research Centre (Pekel et al., 2016) (Fig. 5). Also, on a smaller domain, our assessment proofed good results. Lake Tana and Zeway surface water coverages were estimated to 3095 and 408 km^2^, similar to results extracted from the Global Surface Water dataset with 3132 km^2^ and 434 km^2^, respectively (Pekel et al., 2016). In other studies, the Lake Tana surface water area was estimated to 3041 km^2^ (Ayele and Atlabachew, 2021) and that of Lake Zeway to 418 km^2^ between October 2010 and February 2016 (Asfaw et al., 2020).

The best-performing indices, WI and AWEIsh, were efficient and replicable in Sentinel-2 data using Google Earth Engine computing. Even though they are specifically designed for Landsat sensors (Feyisa et al., 2014; Fisher et al., 2016), they are also effective in producing high-quality maps for large-scale surface water detection using high-resolution Sentinel-2 imagery. Consequently, these methods are useful for policymakers, experts and stakeholders in water resources monitoring, also in the light of missing monitoring efforts of SDG6 for Ethiopia.

Ethiopia’s man-made reservoirs have become critical to the country’s energy and economic development, particularly through hydropower projects like the Grand Ethiopian Renaissance Dam (GERD). The natural lakes of Ethiopia, such as Lake Tana and Lake Zeway, are freshwater lakes and have a considerable amount of outflow. They have both ecological features and cultural significance. They support a variety of ecosystems, supporting aquatic life and contributing to biodiversity. In addition, natural lakes but also wetlands provide ecosystem services such as water filtration and climate regulation. However, changing land use and land cover due to population pressure, water scarcity due to contamination, soil erosion, overgrazed land, and alien species are major problems of African lakes (Singh et al., 2006). In the northern and western areas of the Lake Ziway, high expansion of local and commercial agricultural irrigation is exhibited (Asfaw et al., 2020). Understanding and mitigating anthropogenic and environmental impacts will be critical to achieving a harmonious balance between development and conservation as Ethiopia navigates the complex interaction of man-made and natural water bodies. Water indices per se cannot distinguish between man-made (e.g., constructed wetlands, river diversions, reservoirs) and natural water surfaces. But water indices provide excellent tools for monitoring spatial changes in water coverage. Together with information of long-term land use, water indices could also contribute in the separation of man-made versus natural changes in water surface areas. This would require a sufficiently long time series of land use maps. However, such information is currently not available for Ethiopia.

## Conclusion

This paper demonstrated the potential of water index methods and selected the best-performing indices for large-scale surface water detection using Sentinel-2 data with Google Earth Engine in Ethiopia. Sentinel-2 data processed within Google Earth Engine has immense potential to provide large-scale surface water detection with a very high efficiency, accuracy, and temporal frequency, thereby supporting water resource monitoring and management. The results showed that WI and AWEIsh were the best performers in terms of accuracy indicators for overall accuracy, producer’s accuracy, and user’s accuracy, ranging from 0.94 to 0.97. Applying both indices, the WI and AWEI extracted surface water areas of 82,650 and 86,530 km^2^ respectively. The results confirmed that WI and AWEIsh indices using Sentinel-2 data provide reliable assessments of surface water coverage. Utilizing such indices could substantially improve the monitoring of the country’s reservoirs, which have become critical to the country’s energy and economic development. These best-performing indices could play a critical role in surface water monitoring for water resource planners and decision-makers. They provide accurate, timely, and reliable spatial information to support informed decision-making, planning and development, risk management, and policy formulation related to surface water resources. This would be all the more the case if high-temporal resolution sequences of remote sensing images were available. The application of water indices could also successfully contribute to the achievement of SDG 6 at the regional level, as they are useful for guiding conservation efforts and the sustainable use and management of surface water resources.

However, any approach of surface water detection that only considers remote sensed imagery has difficulties in making future projections of surface water coverage, as images are always from the past. For future projections, for example, if information is needed on climate or land use change effects on surface water resources, dynamic variables that change over time should complement surface water detection. This likely includes hydro-meteorological variables from weather forecasts or climate projections as well as dynamic land use features of land use projections. Such information should be fused with satellite imagery to enable future projections. Machine learning models with data fusion techniques, which have recently shown promising results for accurate surface water detection over a wide range of environmental conditions, are seen as having particular potential for this purpose.

## Data Availability

The data presented in this study are available on request from the corresponding author.
